# The Liver–Testis Axis: Molecular Mechanisms and Clinical Implications

**DOI:** 10.3390/ijms27135873

**Published:** 2026-06-29

**Authors:** Yapeng Zhang, Haoran Xu, Hede Zou, Wei Lin, Wenkang Chen, Jiayou Zhao

**Affiliations:** 1 Institute of Basic Research in Clinical Medicine, China Academy of Chinese Medical Sciences, Beijing 100700, China; zhangyapeng1999@126.com (Y.Z.);; 2 Graduate School of China Academy of Chinese Medical Sciences, Beijing 100700, China; 3 Graduate School of Hebei University of Chinese Medicine, Shijiazhuang 050091, China

**Keywords:** liver–testis axis, metabolic disease, male reproduction, MASLD, review

## Abstract

Metabolic dysfunction-associated steatotic liver disease (MASLD) and male hypogonadism (HG) are prevalent disorders that frequently coexist, suggesting a bidirectional “liver–testis axis” as a potential pathophysiological link. This review explores the mechanistic basis and clinical implications of this axis. Molecularly, metabolically stressed hepatocytes release an altered hepatokine signature—marked by reduced sex hormone-binding globulin (SHBG) and elevated fibroblast growth factor 21 (FGF21)—along with pro-inflammatory cytokines (e.g., interleukin-1 beta (IL-1β), interleukin-6 (IL-6), tumor necrosis factor-alpha (TNF-α)), which enter the systemic circulation. These factors may contribute to the impairment of Leydig cell steroidogenesis, the perturbation of blood–testis barrier integrity, and the disruption of spermatogenesis. Conversely, testicular dysfunction and subsequent testosterone deficiency promote visceral adiposity, worsen insulin resistance and amplify chronic inflammation, thereby accelerating hepatic steatosis and fibrosis. Clinically, these molecular interactions manifest as mutually worsening of MASLD and HG. Thus, the liver–testis axis establishes a framework that reveals the bidirectional crosstalk between hepatic metabolism and gonadal function, providing novel pathophysiological insights into these interconnected conditions.

## 1. Introduction

MASLD has become the most prevalent chronic liver disease worldwide, affecting around 30% of adults [1], with its incidence rising alongside the global epidemics of obesity and type 2 diabetes. Concurrently, the incidence of male HG, a condition closely linked to metabolic dysregulation, has also increased markedly. Plenty of epidemiological evidence shows that these two disorders are not separate. In patients with MASLD, the rate of low testosterone is much higher [2]. On the other hand, patients with hypogonadism have a much higher risk of getting MASLD and progressive liver fibrosis [3]. This strong and long-lasting link suggests that the liver and testes may have a direct pathophysiological interaction. This goes beyond their common metabolic risk factors.

This review aims to propose the “liver–testis axis” as a conceptual framework and its meaning. We will describe its molecular and cellular bases and explain how signals from the liver affect testicular function and how testicular hormones act back to control hepatic metabolism. Then, we will reveal the common factors that connect these two organs. By bringing these ideas together, we aim to offer a primary framework that encourages and enables researchers and clinicians to look beyond traditional disciplinary limits and use a more integrated and whole perspective to understand how metabolic health and reproductive health interact with each other.

## 2. Liver-to-Testis Communication: The Role of Liver-Derived Factors

The liver maintains systemic metabolic balance and communicates with distant organs by releasing many different factors [4]. In people with MASLD, this endocrine function of the liver is severely disrupted. The liver then changes from an organ that maintains balance to a source of harmful substances, which lead to testicular dysfunction [5]. Here, we look into specific mechanisms from the liver based on four key mediators, including sex hormone-binding globulin (SHBG), hepatokines, inflammatory cytokines, and bile acids, which are involved in this crosstalk. We also study how these molecules work and their possible harmful effects on testicular steroidogenesis and spermatogenesis [5].

## 3. SHBG

SHBG is a glycoprotein synthesized predominantly by hepatocytes. It acts as the main high-affinity transporter of testosterone and estradiol in the circulation. It plays an important role in controlling how much of these hormones is available to target tissues, such as the testes and the hypothalamic–pituitary axis [6].

Molecular and cellular mechanistic studies have described the pathways that lead to SHBG downregulation. In vitro experiments with human HepG2 hepatoma cells show that insulin directly suppresses *SHBG* gene expression. This happens through transcriptional repression controlled by the forkhead box A1(FOXA1) and A2 (FOXA2) proteins. This finding suggests a potential mechanistic link between hyperinsulinemia and lower SHBG production in the liver, although whether this relationship operates similarly in primary human hepatocytes and in vivo settings requires further validation [7].

Circulating SHBG levels are much lower in people with MASLD than in healthy controls, showing a clear inverse link between SHBG and hepatic steatosis [8]. Moreover, in male infertility patients with metabolic syndrome (MetS), lower circulating SHBG levels are linked to lower total testosterone and poor sperm morphology. This suggests that SHBG may be a potential indicator of how the liver affects testicular function in the liver–testis axis [9].

Studies have shown that low SHBG is linked to higher liver fat content. Visceral fat and liver fat together play a role in 43% (in women) and 60% (in men) of the association between SHBG and type 2 diabetes. This suggests that lower SHBG may contribute to the development of type 2 diabetes [10].

## 4. FGF21

Fibroblast growth factor 21 (FGF21) is a hormone mainly released by the liver. It is produced in response to metabolic stress, such as nutrient excess or endoplasmic reticulum (ER) stress [11]. However, the potential involvement of FGF21 in the liver–testis axis appears to be complex.

Cellular and animal studies have explained how FGF21 protects germ cell survival. Research on diabetic models shows that FGF21 reduces diabetes-induced germ cell apoptosis. It does this by activating two signaling pathways together: the testicular protein kinase B (AKT) pathway and the adenosine 5‘-monophosphate-activated protein kinase (AMPK)/Sirtuin 1 (SIRT*1*)/peroxisome Proliferator-Activated Receptor Gamma Coactivator 1-Alpha (PGC-1α) pathway [12]. In research on human spermatozoa, FGF21 has been shown to improve sperm motility, increase adenosine triphosphate (ATP) levels, and reduce oxidative stress by acting through the phosphatidylinositol 3-kinase (PI3K)/AKT and mitogen-activated protein kinase (MAPK) signaling pathways [13]. Experiments on primary cultured rat testicular cells demonstrated that FGF21 can dose-dependently inhibit luteinizing hormone(LH)-stimulated androgen production, potentially by suppressing 17α-hydroxylase activity (CYP17A1) [14].

Clinical evidence has shown associations between FGF21, MASLD, and reproductive function. Elevated circulating FGF21 levels are a feature of obesity, insulin resistance, and MASLD in humans. Research has summarized the correlation between high FGF21 levels and these metabolic disorders in a systematic way [15]. Large-scale cross-sectional epidemiological studies have revealed an inverse correlation between serum FGF21 levels and total testosterone concentrations in men, implying an endocrine link between hepatic metabolic stress in MASLD and HG—a connection further corroborated by studies that elucidate the association between hepatic metabolic dysfunction and gonadal function decline [16]. These findings show that FGF21 may act as a key mediator connecting hepatic metabolic disorders in MASLD with reproductive function impairments.

Although FGF21 analogues (such as efruxifermin) improve liver histology and metabolism in patients with metabolic dysfunction-associated steatohepatitis (MASH) [17], their effects on male reproductive endocrine function remain to be revealed. Future randomized controlled trials (RCTs) should therefore clearly set hypogonadism as a predefined endpoint in the study protocol to fill this obvious gap in evidence.

The seemingly contradictory effects of FGF21 on testicular function—protective versus inhibitory—likely reflect fundamental differences in experimental context rather than genuine biological inconsistency. The protective evidence derives primarily from exogenous FGF21 administration in diabetic animal models or direct exposure of human spermatozoa to recombinant FGF21, where supraphysiological or pharmacological doses may activate compensatory metabolic pathways (AKT/AMPK/SIRT1/PGC-1α) that enhance germ cell survival and sperm motility. Conversely, the inhibitory evidence originates from primary cultured rat Leydig cells exposed to FGF21, where dose-dependent suppression of LH-stimulated androgen production suggests a direct steroidogenic inhibitory effect at the cellular level. These divergent outcomes may be attributable to differences in target cell type (germ cells versus Leydig cells), FGF21 concentration and exposure duration (acute pharmacological versus chronic endogenous elevation), and the presence or absence of systemic metabolic milieu. Notably, in vivo studies of endogenous FGF21 elevation in MASLD have not systematically dissociated the hormone’s direct testicular effects from its systemic metabolic improvements; thus, whether the observed inverse correlation between serum FGF21 and testosterone in epidemiological studies reflects direct Leydig cell inhibition, indirect metabolic benefits, or both remains unresolved.

Elevated circulating FGF21 levels are commonly observed in patients with MASLD, and this phenomenon can be explained by several mechanisms. Hepatic upregulation of FGF21 represents an adaptive response to metabolic stress, aiming to enhance systemic insulin sensitivity and facilitate lipid oxidation. Its impacts on the testes are merely incidental or secondary effects. As a protective hepatokine, elevated FGF21 primarily indicates the presence of metabolic disorders rather than actively inducing reproductive dysfunction. On the other hand, under chronic metabolic stress accompanied by potential receptor desensitization, sustained hypersecretion of FGF21 directly impairs Leydig cell function and triggers hypogonadism. In this context, FGF21 acts as a key molecular mediator of pathological crosstalk between the liver and testes. During the early stage of metabolic disturbance, FGF21 exerts compensatory and protective effects. However, prolonged exposure and dysregulation of downstream signaling pathways gradually shift its role toward pathogenicity. This temporal pattern resembles the transition of endoplasmic reticulum (ER) stress from physiological adaptation to pathological injury. Current studies have demonstrated that FGF21 analogues can improve liver histology, yet reproductive function parameters have not been included as study endpoints. Additionally, there is a lack of longitudinal investigations tracking the dynamic changes in FGF21 and testosterone levels throughout MASLD progression. These critical research gaps need to be addressed to clarify the exact role of FGF21 in the liver–testis axis: whether it merely serves as a biomarker for comorbid disorders, an adaptive signal, or a causative pathogenic mediator.

### 4.1. Inflammatory Cytokines

Chronic inflammation is a hallmark of progressive MASLD [18]. It is considered a contributor of disease progression, promoting the transition from simple steatosis to MASH, liver fibrosis, cirrhosis, and, finally, hepatocellular carcinoma (HCC). This widespread inflammatory environment is also linked to the development of reproductive problems, such as male HG and impaired spermatogenesis [19]. Inflammatory cytokines have been proposed as potential mediators of the onset and progression of MASLD.

Accumulating evidence from both animal and clinical studies indicates that targeting pro-inflammatory cytokines represents a promising therapeutic strategy to ameliorate the gonadal dysfunction associated with metabolic diseases [20,21]. Notably, the liver constitutes a major source of cytokine production. This is largely attributable to hepatic immune cells, particularly Kupffer cells—the resident tissue macrophages of the liver—which release substantial quantities of cytokines. These signaling molecules can exert local effects within the liver or enter the systemic circulation, raising the possibility of distal organ effects, including in the testes, although direct evidence for physiologically relevant liver-to-testis cytokine transmission in vivo remains limited [22]. In addition to immune cells, hepatocytes themselves may produce certain cytokines under metabolic stress [23]. Although the specific range of hepatocyte-derived cytokines linked to testicular inflammation is not yet fully characterized, circulating levels of key pro-inflammatory cytokines made in the liver rise sharply when the condition progresses from simple steatosis to MASH. These cytokines are hypothesized to act as critical drivers of the related reproductive dysfunction [24,25].

**Figure 1:**
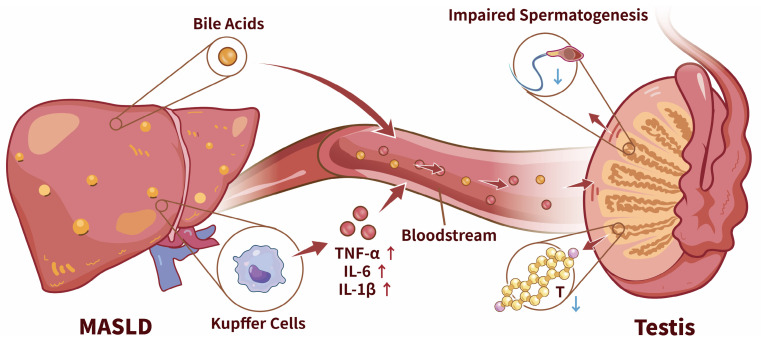
Liver-to-testis communication: inflammatory cytokines and bile acid dysregulation. In MASLD, hepatic Kupffer cells become activated and release the pro-inflammatory cytokines TNF-α, IL-1β, and IL-6 into the systemic circulation. Upon reaching the testes, these cytokines directly downregulate the key steroidogenic proteins StAR and CYP17A1 in Leydig cells, thereby reducing T biosynthesis. Concurrently, hepatic bile acid dysregulation exacerbates systemic and testicular oxidative stress as well as inflammation, which impairs the development and functional integrity of germ cells and ultimately culminates in impaired spermatogenesis. MASLD, metabolic dysfunction-associated steatotic liver disease; TNF-α, tumor necrosis factor-α; IL-1β, interleukin-1β; IL-6, interleukin-6; StAR, steroidogenic acute regulatory protein; CYP17A1, cytochrome P450 17A1; T, testosterone. Green arrows indicate normal secretion levels or promotion; red arrows indicate abnormal levels.

HG in men with MASLD has been hypothesized to involve, in part, cytokine-mediated suppression of testicular steroidogenesis. This pathway is backed by a growing amount of in vitro evidence. In studies using the mouse TM3 Leydig cell line, exposure to TNF-α or IL-1β dose-dependently suppressed testosterone production and significantly downregulated key steroidogenic enzymes [26]. Similar observations were reported in primary mouse Leydig cells, where TNF-α and IL-1β suppress cAMP-stimulated testosterone secretion by downregulating key steroidogenic enzymes, including *CYP17A1*, at both the mRNA and protein levels [27]. At the molecular level, the inhibition of *Cyp17a1* gene transcription is a key mechanism that allows TNF-α to suppress testosterone synthesis. This process is mediated through the protein kinase C (PKC) signaling pathway, which offers early insight into how inflammation impairs Leydig cell function [28]. This in vitro evidence provides a mechanistic basis for how cytokine dysregulation in MASLD directly impairs testicular endocrine function (Figure 1).

The evidence summarized above positions pro-inflammatory cytokines, particularly TNF-α, IL-1β, and IL-6, as plausible mediators in the proposed liver–testis communication framework, with in vitro studies consistently demonstrating their capacity to suppress Leydig cell steroidogenesis through transcriptional downregulation of CYP17A1 and related enzymes. However, several critical uncertainties constrain mechanistic interpretation. First, the majority of evidence derives from isolated Leydig cell cultures or ex vivo preparations, where cytokine concentrations and exposure durations may not replicate the chronic, low-grade inflammatory milieu characteristic of MASLD. Second, the hepatic origin of circulating cytokines reaching the testes cannot be definitively distinguished from adipose tissue, macrophages, or other systemic sources, complicating the attribution of testicular effects to liver-derived signals specifically. Third, cytokine signaling intersects with other hepatic stressors discussed in preceding sections: TNF-α shares PKC-mediated pathways with insulin resistance-induced metabolic disruption, while IL-6 signaling converges on Janus kinase-signal transducer and activator of transcription (JAK-STAT) and mitogen-activated protein kinase (MAPK) cascades that partially overlap with FGF21 downstream effects. This mechanistic convergence raises the possibility that cytokines, hepatokines, and metabolic stressors may act synergistically or redundantly to impair testicular function, although whether such multi-signal integration occurs in vivo and which mediator dominates under specific pathophysiological conditions remain entirely speculative. Finally, the blood–testis barrier, discussed in subsequent sections regarding lipotoxicity and oxidative stress, likely modulates cytokine access to the adluminal compartment, introducing an additional anatomical variable that has received insufficient experimental attention. Thus, while cytokine-mediated suppression of testicular steroidogenesis represents a coherent in vitro finding, its elevation to a validated component of liver–testis axis signaling awaits organ-specific tracing studies and longitudinal in vivo models.

### 4.2. Bile Acids

Bile acids, classically known for their role in dietary lipid absorption, are now recognized as potent signaling molecules that regulate systemic metabolic homeostasis through activation of receptors such as the farnesoid X receptor (FXR) and the G protein-coupled bile acid receptor 1 (TGR5). MASLD is often linked to changes in bile acid composition and pool size, a condition called bile acid dysregulation. This metabolic disturbance is associated with the development of extrahepatic complications, including testicular dysfunction [29,30].

Evidence from in vitro and animal models shows that bile acids may influence testicular function, and studies have confirmed the expression of FXR in Leydig cells of both rodents and humans. In the mouse Leydig tumor cell line (MLTC-1), treatment with specific FXR agonists reduced the expression of key steroidogenic enzymes (including StAR and Cyp11a1), leading to a marked decrease in cAMP-stimulated testosterone production [31,32]. Furthermore, studies on animal models of diet-induced MASLD have shown that the resulting changes in the bile acid pool are linked to testicular inflammation and oxidative stress. Mice with specific genetic modifications to bile acid synthesis or signaling—such as *Fxr* knockout mice—develop more severe testicular degeneration when fed a high-fat diet, suggesting that intact FXR signaling may be relevant to testicular homeostasis under metabolic stress in this model [33,34].

The role of TGR5 signaling in the testes is a newly emerging research area. Although the presence of TGR5 in human spermatozoa has been reported, its functional role in steroidogenesis remains unclear. Some in vitro studies suggest that TGR5 activation may affect mitochondrial function and energy metabolism in other steroidogenic tissues, implying a potential—but not yet fully defined—role in Leydig cell function that deserves further exploration [35,36].

Translating these findings to clinical practice, observational studies in men have begun to show an association between bile acid metabolism and reproductive hormones. Patients with advanced MASLD or primary biliary cholangitis—disorders marked by impaired bile acid homeostasis—often exhibit altered serum bile acid profiles and a higher prevalence of HG [37,38].

Bile acid dysregulation in MASLD introduces another layer of potential metabolic signaling to testicular function, operating through nuclear receptor FXR and membrane receptor TGR5 pathways that partially overlap with, yet remain distinct from, the hepatokine and cytokine mechanisms discussed above. Unlike SHBG or FGF21, which primarily modulate steroidogenesis via endocrine or metabolic signaling, bile acids appear to exert more direct transcriptional effects on steroidogenic enzyme expression, with FXR activation in Leydig cells associated with reduced StAR and CYP11A1 expression. However, the functional interpretation of these findings remains complicated: *Fxr* knockout exacerbates testicular degeneration under metabolic stress, suggesting that intact bile acid–FXR signaling may be necessary for testicular homeostasis, yet pharmacological FXR agonism suppresses testosterone production in vitro. This apparent paradox—whether FXR signaling is protective or inhibitory—may reflect differences between acute pharmacological stimulation and chronic adaptive responses or between tumor cell lines and physiological conditions. TGR5 adds further complexity, as its expression in spermatozoa and potential effects on mitochondrial energetics introduce a spatially distinct mechanism from the nuclear FXR pathway. Whether these bile acid receptors mediate true liver-to-testis endocrine signaling, or whether testicular bile acid exposure primarily reflects local synthesis or systemic overflow from hepatic dysregulation, has not been established. Collectively, the evidence positions bile acids as candidate mediators in liver–testis communication, but their precise role relative to inflammatory cytokines and lipotoxic insults—particularly in determining whether multiple hepatic stressors converge on common testicular endpoints or target distinct cellular compartments—requires integrated multi-omic and interorgan tracing approaches.

However, these therapeutic implications have not yet been applied in clinical practice because the effects of pharmacological FXR agonists or bile acid sequestrants on male reproductive function in MASLD patients have not been systematically evaluated; this represents a key gap for future research [39]. Thus, further research is needed to confirm that bile acids contribute to testicular dysfunction (Figure 1).

The liver-secreted mediators mentioned above, including decreased SHBG and increased FGF21 and pro-inflammatory cytokines, share a core pathophysiological feature: metabolic stress remodels hepatic secretory patterns and impairs testicular function via independent but partially intersecting mechanisms.

SHBG reduction primarily limits testosterone bioavailability and may disrupt feedback regulation at the hypothalamic–pituitary level. FGF21 exerts seemingly context-dependent effects, with in vitro and ex vivo data suggesting both protective and inhibitory actions on testicular cells. Inflammatory cytokines, particularly TNF-α and IL-1β, appear to suppress steroidogenic enzyme expression through PKC-mediated transcriptional repression. Notably, these mediators do not operate in isolation. Hyperinsulinemia, a hallmark of MASLD, simultaneously suppresses SHBG production and promotes systemic inflammation, raising the possibility that reduced SHBG and elevated cytokines may act synergistically to impair Leydig cell function. Similarly, FGF21 and IL-6 share downstream signaling components, including AMPK and MAPK pathways, suggesting potential crosstalk between hepatokine and cytokine signaling in the testes. Nevertheless, it remains unclear whether these in vitro findings and epidemiological correlations indicate genuine pathway crosstalk in vivo or merely reflect separate parallel responses to systemic metabolic disorders. Furthermore, the relative contribution of each mediator to testicular impairment in MASLD patients—versus their collective effect or potential compensatory interactions—has not been systematically evaluated.

### 4.3. Testis-to-Liver Feedback: The Hepatic Impact of Gonadal Hormones

While the liver-to-testis axis underscores the liver’s endocrine role in male reproductive health, reciprocal communication from the testes to the liver is equally critical. Sex hormones—mainly testosterone and estrogen—exert significant effects on hepatic lipid metabolism, insulin sensitivity, and inflammatory processes, thereby establishing a complete feedback loop within the liver–testis axis [40,41].

### 4.4. Testosterone

Testosterone, the primary male sex hormone, plays a key protective role in maintaining hepatic metabolic homeostasis. Cellular and molecular studies have shown that testosterone signaling via the androgen receptor (AR) in hepatocytes promotes fatty acid β-oxidation and suppresses de novo lipogenesis by downregulating key lipogenic transcription factors, such as sterol regulatory element-binding protein 1c (SREBP-1c) [42,43,44].

This is supported by strong evidence from animal models. Orchiectomy in male rodents consistently leads to the rapid development of hepatic steatosis and insulin resistance, while testosterone replacement in these models or in male mice with diet-induced obesity effectively reverses lipid accumulation and reduces hepatic inflammation [45]. The clinical relevance of these findings is clear. Numerous epidemiological studies have demonstrated a strong, independent inverse association between low serum testosterone levels and the presence and severity of MASLD in men—even after adjusting for age and adiposity [46,47]. Furthermore, interventional clinical trials have begun to show that testosterone replacement therapy (TRT) in hypogonadal men—particularly those with confirmed MASLD—can lead to significant improvements in liver fat content (assessed by non-invasive imaging) and serum liver enzyme levels. This positions testosterone not merely as a biomarker but also as a potential modulator of liver pathology [48,49]. These findings underscore the critical role of testosterone in preserving hepatic metabolic health and provide a robust rationale for exploring testosterone-related interventions (e.g., TRT) as a potential strategy for managing MASLD in hypogonadal men. They also highlight the clinical significance of monitoring testosterone levels in male patients with liver metabolic disorders.

**Figure 2:**
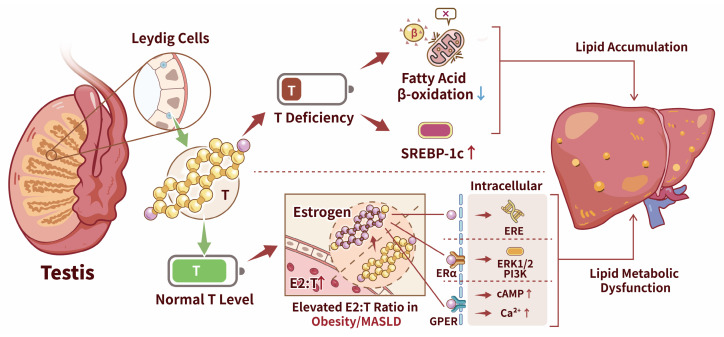
Testis-to-liver: The hepatic impact of gonadal hormones. T secreted by the testes activates the AR in hepatocytes, thereby promoting fatty acid β-oxidation and inhibiting SREBP-1c to counteract hepatic steatosis. Conversely, testosterone deficiency impairs this protective effect and exacerbates the progression of hepatic lipid deposition and inflammation. In obesity and MASLD, increased aromatase activity in adipose tissue elevates the estrogen-to-androgen ratio. The elevated estrogens directly regulate a large repertoire of hepatic genes—particularly those implicated in lipid metabolism—via the nuclear ERα, while activating the ERK1/2 and PI3K signaling pathways through membrane-localized actions. Concurrently, the membrane-associated GPER contributes to this regulatory network via cAMP and Ca^2+^ signaling cascades. The synergistic effects of these signaling pathways perturb the hepatic lipid metabolic program, thereby exacerbating hepatic lipid deposition and metabolic dysregulation. AR, androgen receptor; SREBP-1c, sterol regulatory element-binding protein 1c; MASLD, metabolic dysfunction-associated steatotic liver disease; ERα, estrogen receptor α; ERK1/2; extracellular signal-regulated kinase 1/2; PI3K, phosphatidylinositol 3-kinase; GPER, G protein-coupled estrogen receptor; cAMP, cyclic adenosine monophosphate; Ca^2+^, calcium ion. Green arrows indicate normal secretion levels or promotion; red arrows indicate abnormal levels.

However, current clinical studies investigating the association between testosterone and MASLD have several limitations. Most epidemiological studies demonstrating this correlation adopt a cross-sectional or case–control design, which cannot determine the temporal sequence of events or establish causality. Low testosterone and MASLD share multiple common risk factors, including visceral obesity, insulin resistance, abnormal SHBG levels, type 2 diabetes mellitus, and concomitant medications such as statins and glucocorticoids. These factors can independently affect both hepatic and gonadal function. Although some studies have adjusted for confounders such as age and body mass index, residual biases may still arise from visceral fat distribution, the severity of insulin resistance and lifestyle-related factors. Furthermore, observational studies alone cannot rule out reverse causality. Hepatic metabolic disorders may suppress testosterone synthesis via inflammatory and lipotoxic mechanisms. While interventional trials on TRT have yielded promising results, they are generally hampered by small sample sizes, short follow-up periods and the absence of liver histological endpoints. Accordingly, it remains unclear whether testosterone modulation directly ameliorates the pathological progression of MASLD or merely leads to concurrent improvements in metabolic risk factors. In conclusion, the clinical view that testosterone acts as a regulator of liver pathology is still preliminary. The bidirectional relationship between testicular function and hepatic metabolism in humans needs to be verified by large-scale prospective cohort studies and adequately powered RCTs with predefined liver-related outcomes (Figure 2).

### 4.5. Estrogen:Androgen (E2:T) Ratio

The balance between androgens and estrogens is equally critical for hepatic metabolism [44]. As a key receptor, estrogen receptor alphaERα directly regulates over 1000 sex-biased hepatic genes—with a particular enrichment in lipid metabolism pathways—either by binding to estrogen response elements (EREs) in the nucleus or by activating the extracellular signal-regulated kinase 1 and 2 (ERK1/2) and PI3K signaling pathways through membrane localization. Meanwhile, the G protein-coupled estrogen receptor (GPER) contributes to this regulatory network via cyclic adenosine monophosphate (cAMP) and Ca^2+^ signaling cascades. Together, these two receptors enable the precise modulation of fatty acid, triglyceride (TG), and cholesterol metabolism [50]. In men, a substantial portion of circulating estrogens is derived from the aromatization of androgens in adipose tissue [51]. In the context of obesity and MASLD, adipose tissue expansion leads to increased aromatase activity, which, in turn, elevates estrogen levels and consequently alters the estrogen–androgen ratio [52]. Experimental models provide insights into the dual role of estrogen, where physiological estrogen signaling via ERα is generally hepatoprotective and promotes beneficial lipid metabolism [53]. However, supraphysiological estrogen levels or dysregulated signaling observed in obese males can exacerbate hepatic lipid deposition [54]. Male mice with global or hepatocyte-specific deletion of ERα develop spontaneous hepatic steatosis, which underscores the protective role of this hormone under physiological conditions [55]. In clinical practice, the significance of this balance is reflected in conditions involving estrogen excess—such as in men receiving estrogen therapy or diagnosed with aromatase excess syndromes. These patients often present with gynecomastia and fatty liver disease, which highlights the detrimental hepatic effects of an imbalanced estrogen–androgen ratio in males [56,57]. This complex interplay indicates that the relative excess of estrogen coupled with testosterone deficiency—a common condition in obese male patients with MASLD—creates a synergistic adverse metabolic environment for the liver (Figure 2).

### 4.6. Common Regulators of Hepatic and Testicular Dysfunction

The concurrent dysfunction of the liver and testes in metabolic diseases is not merely coincidental but rather driven by shared systemic pathophysiological processes [58]. These common regulators act as upstream drivers that simultaneously disrupt homeostasis in both organs and establish a self-reinforcing pathological cycle within the liver–testis axis.

### 4.7. Insulin Resistance (IR)

Insulin resistance represents a pathophysiological bridge linking hepatic steatosis to testicular dysfunction. At the molecular level, impaired insulin signaling in hepatocytes promotes de novo lipogenesis and reduces fatty acid oxidation, while in Leydig cells, it disrupts the insulin-mediated potentiation of LH-stimulated testosterone production, a process considered important for normal steroidogenesis [59,60]. Animal models provide direct causal evidence: mice with liver-specific insulin receptor knockout (LIRKO) develop severe insulin resistance and dyslipidemia, but male mice with diet-induced IR consistently exhibit both hepatic steatosis and low testosterone levels, with the latter being reversible upon improvement in insulin sensitivity [61,62]. In the clinical realm, epidemiological data confirm that hyperinsulinemia is an independent predictor of low serum testosterone in men; conversely, men with HG exhibit a higher prevalence of IR and MASLD, highlighting the bidirectional nature of this relationship within the axis [63,64].

Mechanistically, the divergence in insulin signaling between these two organs hinges on differential IRS isoform expression and downstream metabolic partitioning [65,66]. In hepatocytes, insulin resistance manifests as impaired hepatic IRS–PI3K/AKT signaling, which fails to suppress FOXO1-driven gluconeogenesis while paradoxically preserving lipogenic pathways via mTORC1/SREBP-1c activation, thereby accelerating hepatic steatogenesis [67]. In Leydig cells, which express IRS-2, insulin resistance disrupts insulin-dependent PI3K/AKT signaling, impairing testosterone synthesis and attenuating the steroidogenic response to LH stimulation [26,68,69]. Specifically, because insulin signaling normally primes LH-activated cAMP/PKA responses, its disruption diminishes PKA-mediated phosphorylation, reducing the functional stability and mitochondrial translocation of StAR, while impaired cAMP/PKA crosstalk impairs CYP17A1 enzymatic activity [70,71,72]. Concurrently, unchecked GSK-3*β* activation destabilizes β-catenin, potentially suppressing StAR gene transcription and further attenuating steroidogenic capacity [73]. This organ-specific molecular divergence highlights the pleiotropic and directionally distinct consequences of systemic insulin resistance within the liver–testis axis.

### 4.8. Systemic Inflammation and Oxidative Stress

Chronic, low-grade inflammation and resultant oxidative stress serve as a common destructive thread weaving through the pathophysiology of both MASLD and HG [74,75]. Cellular studies demonstrate that pro-inflammatory cytokines and reactive oxygen species (ROS) can directly inhibit steroidogenic gene expression in Leydig cells while simultaneously activating pro-fibrotic pathways in hepatic stellate cells, illustrating a shared mechanism of cellular injury. This is mirrored in vivo: animal models of MASLD show elevated levels of systemic and testicular inflammatory markers (e.g., TNF-α, IL-6) and oxidative damage, which correlate with the severity of both hepatic steatosis and testicular apoptosis [76,77]. Clinical evidence solidifies this link, as men with MASLD display elevated circulating levels of inflammatory markers like high-sensitivity c-reactive protein (hs-CRP) and IL-6, which are independently and inversely correlated with testosterone concentrations, positioning systemic inflammation as a key mediator of gonadal dysfunction in metabolic disease [78,79].

At the molecular level, the crosstalk between metabolic stress and inflammatory activation converges on the NOD-like receptor family pyrin domain-containing 3 (NLRP3) inflammasome, which is functionally expressed in hepatic Kupffer cells and testicular Sertoli cells, while adjacent testicular macrophages deploy NLRP3-derived IL-1β to modulate Leydig cell steroidogenesis [80,81,82]. In the steatotic liver, saturated fatty acid-induced mitochondrial ROS, alongside lysosomal destabilization, triggers NLRP3 assembly, driving caspase-1-dependent maturation of IL-1β and IL-18 that propagate local and systemic inflammation [83,84]. In the testes, hyperglycemia- and lipotoxicity-associated oxidative stress activate the testicular NLRP3 inflammasome, thereby impairing Leydig cell steroidogenesis and disrupting Sertoli cell tight junction integrity [85,86,87]. Concurrently, unresolved endoplasmic reticulum (ER) stress operates as a parallel amplifier in both organs: in hepatocytes, IRE1α/XBP1 signaling promotes de novo lipogenesis and pro-inflammatory transcription, whereas in Leydig cells, PERK/eIF2α/ATF4 activation suppresses the expression of StAR and 3β-hydroxysteroid dehydrogenase (3β-HSD), linking ER stress to impaired androgen synthesis [88,89,90]. Furthermore, ROS-driven disruption of tight junction proteins—such as occludin and claudin-11—compromises the blood–testis barrier, facilitating the intra-testicular infiltration of inflammatory mediators and creating a self-perpetuating local inflammatory microenvironment [91,92,93,94,95,96,97].

### 4.9. Lipotoxicity

Lipotoxicity, the pathological accumulation of lipid intermediates like diacylglycerols (DAGs) and ceramides in non-adipose tissues, is an effector of cellular dysfunction in both organs [98]. Mechanistic studies show that in hepatocytes, ceramides promote insulin resistance and apoptosis, while in Leydig cells, they disrupt mitochondrial function and directly suppress steroidogenic enzyme activity, creating a parallel pathway of cellular failure [99,100,101]. Animal studies confirm that diets high in saturated fat lead to the accumulation of toxic lipids in both the liver and testes, and interventions that reduce ceramide synthesis can ameliorate dysfunction in both organs, highlighting a shared pathogenic pathway. Supporting clinical data indicate that circulating levels of certain ceramide species are elevated in men with MASLD and are negatively associated with serum testosterone levels, providing a potential mechanistic lipid signature for the liver–testis axis [102].

The divergent lipotoxic fates of free fatty acids (FFAs) in hepatocytes versus Leydig cells further illuminate the organ-specific details within this axis [103,104]. In the liver, excessive FFA influx promotes the accumulation of DAG*s*, which activate PKCε, leading to serine phosphorylation of IRS-1/2 and subsequent blockade of PI3K/AKT signaling [105,106,107,108]. This DAG-PKC axis constitutes a central mechanism linking hepatic lipid overload to selective insulin resistance. In contrast, within Leydig cells, saturated FFAs can be diverted toward ceramide generation via both de novo synthesis and salvage pathways, with the resulting ceramide accumulation inhibiting steroidogenesis and promoting apoptosis [109,110,111,112]. Accumulated ceramides disrupt mitochondrial electron transport chain complex I, III, and IV activity, precipitating ROS burst and collapse of mitochondrial membrane potential (ΔΨm), which curtails the ATP-dependent cholesterol transport mediated by StAR [113,114,115,116,117]. Moreover, ceramide-activated protein phosphatase 2A (PP2A) dephosphorylates and inactivates AKT while simultaneously promoting the nuclear translocation of FOXO3a, a transcriptional suppressor of *StAR* that curtails testosterone synthesis [118,119]. Notably, lipotoxicity also impairs the blood–testis barrier by disrupting F-actin organization and tight junction protein expression in Sertoli cells, permitting the leakage of toxic lipid intermediates and inflammatory cytokines into the adluminal compartment, thereby amplifying germ cell apoptosis and compromising spermatogenic integrity [95,97,120,121,122].

Lipotoxicity introduces a distinct mechanistic dimension to the proposed liver–testis axis, operating not through receptor-mediated signal transduction but via direct intracellular accumulation of lipid intermediates—particularly ceramides and (DAGs)—that disrupt mitochondrial energetics and insulin signaling in both organs. In hepatocytes, the DAG-PKCε axis drives selective insulin resistance, paradoxically preserving lipogenic capacity while impairing glucose homeostasis; in Leydig cells, ceramide accumulation appears to suppress StAR-mediated cholesterol transport and CYP17A1 activity through convergent effects on mitochondrial membrane potential and AKT-FOXO3a signaling. This organ-specific divergence in lipotoxic fate—hepatic DAGs promoting steatogenesis versus testicular ceramides inhibiting steroidogenesis—suggests that saturated fatty acid overload may trigger directionally opposite metabolic consequences depending on tissue-specific lipid partitioning and enzymatic repertoire. Notably, these lipotoxic pathways intersect with mechanisms discussed in preceding sections: ceramide-activated PP2A dephosphorylates AKT, overlapping with insulin resistance-mediated signaling disruption; ROS generation from mitochondrial complex dysfunction amplifies NLRP3 inflammasome activation, as described in the Inflammatory Cytokines section; and ER stress, detailed below, represents a parallel consequence of lipotoxic insult in both organs. Furthermore, lipotoxicity compromises blood–testis barrier integrity through F-actin disruption in Sertoli cells, potentially facilitating the intra-testicular penetration of inflammatory mediators and bile acid derivatives discussed earlier. This creates a hypothetical feed-forward loop wherein hepatic steatosis-driven lipotoxicity not only directly impairs Leydig cell function but also renders the testicular microenvironment more susceptible to cytokine and bile acid insults. However, direct evidence that hepatic-derived lipid intermediates reach the testes in concentrations sufficient to elicit these effects in vivo is currently lacking; circulating ceramide levels correlate with both MASLD severity and hypogonadism, yet whether this association reflects directed interorgan transport, systemic spillover, or independent adipose–hepatic–testicular production remains unresolved. The lipotoxicity framework thus complements, rather than supersedes, the hepatokine, cytokine, and bile acid hypotheses, raising the broader question of whether multiple hepatic stressors converge on shared testicular endpoints through distinct proximal mechanisms or whether their co-occurrence merely reflects the systemic nature of metabolic disease.

## 5. Conclusions and Future Perspectives

Traditionally, the liver, serving as the body’s metabolic hub, and the testes, functioning as the primary site of androgen production and spermatogenesis, have been studied within distinct medical specialties. However, modern research increasingly focuses on organ crosstalk, or “axes,” providing a novel systems biology perspective for understanding complex diseases. Well-established concepts like the “cardiohepatic axis”—where hepatic inflammation and fibrosis directly promote cardiac dysfunction [123]—or the “gut–liver axis”—where intestinal dysbiosis acts as a key driver of hepatic injury [124]—have profoundly reshaped our management strategies for related conditions. This raises a pivotal question: does a comparable “liver–testis axis” exist?

We hypothesize that the liver and testes engage in bidirectional communication via a network of hormones, inflammatory cytokines and metabolites. Under metabolic stress, a steatotic liver becomes an active “endocrine disruptor,” secreting dysregulated hepatokines (e.g., decreased SHBG) [125] and pro-inflammatory mediators (e.g., IL-1β, IL-6, TNF-α) that can systemically impair testicular endocrine and reproductive functions [126]. This “cytokine spillover” effect, a mechanism also observed in other organ axes, can disrupt testicular homeostasis. Simultaneously, functionally compromised testes, due to reduced testosterone production, fail to adequately maintain lean body mass, appropriate fat distribution, and insulin sensitivity [127], thereby reciprocally exacerbating the liver’s metabolic burden and inflammation and creating a self-reinforcing vicious cycle. Furthermore, shared cellular mechanisms—including mitochondrial dysfunction and endoplasmic reticulum stress [128] and immune cell reprogramming [129]—provide the fundamental pathobiological underpinnings that fortify this axis. The concept is reinforced by studies showing that processes like clonal hematopoiesis and bone marrow reprogramming contribute to systemic inflammation [130] in cardiometabolic disease, suggesting similar processes could link hepatic and testicular dysfunction.

The epidemiological associations between MASLD and male hypogonadism cited throughout this review warrant cautious interpretation in light of substantial methodological constraints. The majority of available clinical evidence derives from cross-sectional or retrospective case–control studies, which are inherently limited in establishing temporal sequence and causal direction. Whether MASLD precedes and contributes to testicular dysfunction, whether hypogonadism accelerates hepatic metabolic deterioration, or whether both conditions arise independently from common upstream determinants remains unresolved.

Multiple confounding variables must be explicitly considered in future investigations. Obesity, particularly visceral adiposity, is a well-established shared risk factor for both MASLD and low testosterone; aromatase activity in adipose tissue directly alters the estrogen-to-androgen ratio. Insulin resistance, a central pathophysiological feature of metabolic disease, impairs hepatic lipid metabolism while simultaneously disrupting Leydig cell steroidogenesis, yet few studies have employed methodologies capable of distinguishing hepatic from systemic insulin sensitivity. Reduced SHBG, frequently observed in MASLD, may reflect impaired hepatic synthetic capacity or hyperinsulinemia rather than functioning exclusively as a mediator of liver–testis organ crosstalk; this complicates the interpretation of total testosterone measurements in this population. Type 2 diabetes and its pharmacological management may independently affect both hepatic histology and gonadal function, and medication histories have been inconsistently documented in existing studies. Furthermore, alcohol consumption, physical activity, and sleep disturbances all influence testosterone levels and hepatic fat content but have rarely been comprehensively assessed.

The aforementioned considerations indicate that the existing evidence alone is insufficient to establish a definitive bidirectional regulatory relationship between the two organs. Future research priorities should include: prospective cohort studies with serial measurements of hepatic fat content, hormone profiles, and inflammatory markers to clarify temporal sequence and Mendelian randomization analyses utilizing genetic variants associated with testosterone, SHBG, and MASLD susceptibility to infer causality free from confounding, thereby further elucidating the clinical significance of the liver–testis axis.

In conclusion, evidence from clinical, animal, and cellular studies is consistent with the hypothesis that the liver and testes may engage in bidirectional communication, a concept herein referred to as the “liver–testis axis.” We have revealed how liver-derived factors, such as hepatokines (SHBG, FGF21), pro-inflammatory cytokines, and bile acids, act as endocrine mediators that impair testicular steroidogenesis and spermatogenesis in the context of MASLD. Conversely, the ensuing HG and testosterone deficiency feedback to exacerbate hepatic steatosis, inflammation, and insulin resistance, creating a self-perpetuating vicious cycle. This pathological crosstalk is further amplified by shared upstream drivers, including systemic insulin resistance, chronic inflammation, and lipotoxicity.

These findings establish the liver–testis axis as a novel therapeutic framework for simultaneously alleviating metabolic diseases and reproductive dysfunction in affected males, with future research warranted to discover the precise molecular circuits governing this organ crosstalk.

## Data Availability

No new data were created or analyzed in this study. Data sharing is not applicable to this article. All figures in this manuscript were created using Adobe Illustrator 2023.
